# Midgut protrusion, rotation, and retraction induced by temporal alteration in differential growth

**DOI:** 10.1007/s10237-025-01999-8

**Published:** 2025-09-13

**Authors:** Michina Saiki, Hironori Takeda, Yuto Kawabata, Shunichi Ishida, Yohsuke Imai

**Affiliations:** 1 Graduate School of Engineering, Kobe University, 1-1 Rokkodai-cho, Nada-ku, Kobe, Hyogo 657-8501 Japan; 2 Graduate School of Engineering, Kyoto University, 53 Shogoin-Kawahara-cho, Sakyo, Kyoto 606-8507 Japan; 3 Institute for Life and Medical Sciences, Kyoto University, 53 Shogoin-Kawahara-cho, Sakyo, Kyoto 606-8507 Japan

**Keywords:** Midgut morphogenesis, Physiological umbilical herniation, Continuum mechanics, Isogeometric analysis

## Abstract

Herniation, rotation, looping, and retraction of the midgut occur sequentially during midgut morphogenesis. Recent studies have demonstrated the importance of mechanical forces arising from the differential growth between the midgut and mesentery in the formation of small intestinal loops. However, the roles of mechanics and differential growth in the overall process remain unclear. In this study, we developed a computational model of midgut morphogenesis based on continuum mechanics. We showed that the protrusion, rotation, and retraction of the midgut can emerge sequentially because of temporal changes in differential growth. The midgut was modeled as a hyperelastic tube with a Gaussian shape. The differential growth of the midgut and mesentery was modeled by the spatial variation in spontaneous plastic deformation. The hyperelastic tube developed a protrusion by compression-induced deformation, suggesting that other external forces are not necessary for midgut herniation prior to rotation. Appropriate differential growth induced a $90^{\circ }$ rotation of the tube. A less-growing mesentery attempts to face inward to minimize the tensile forces, which causes tube twisting and results in midgut rotation. Excess differential growth may cause the retraction of the midgut before the formation of small intestinal loops. The results of this study will serve as reference in future studies on embryology and tissue engineering.

## Introduction

**Fig. 1:**
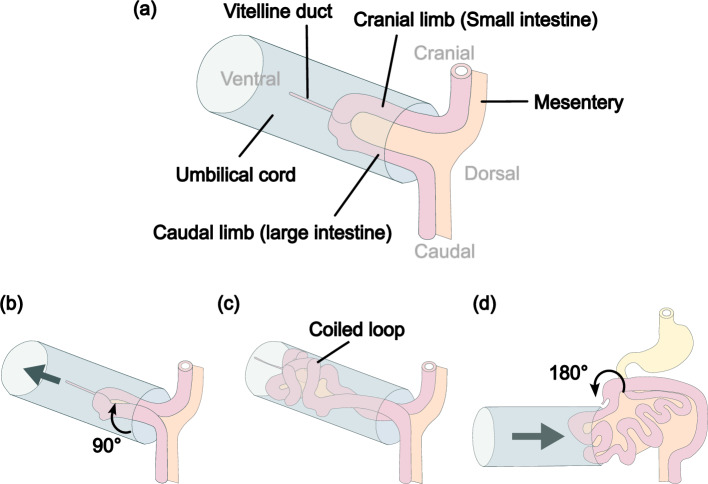
Midgut morphogenesis. **a** Herniation: U-shaped midgut protrudes in the umbilical cord. **b** Rotation: the midgut rotates counterclockwise, as viewed from the ventral side. **c** Looping: small intestinal loops are formed. **d** Retraction: the midgut retracts to the abdominal cavity with $180^{\circ }$ rotation

Midgut morphogenesis is divided into four sequential processes: herniation, rotation, looping, and retraction (Mall 1898; Frazer and Robbins 1915). The midgut, connected to the dorsal mesentery (Hikspoors et al. 2019), forms a U-shaped midgut. The midgut protrudes into the extraembryonic coelom, in a process known as physiological umbilical herniation (Fig. 1a). Next, the midgut rotates $90^{\circ }$ counterclockwise from the ventral side (Fig. 1b), where the cranial limb (small intestine) moves to the right side and the caudal limb (large intestine) moves to the left side. The cranial limb of the midgut grows rapidly and forms small intestinal loops resembling coils (Fig. 1c). Finally, the midgut retracts into the abdominal cavity with a further $180^{\circ }$ rotation (Fig. 1d).

Malrotation, including inadequate or reversed rotation, can lead to abnormal gut arrangement (Strouse 2004; Lampl et al. 2009; Martin and Shaw-Smith 2010; Nehra and Goldstein 2011). Therefore, the mechanism of midgut rotation has been the focus of substantial research (Grzymkowski et al. 2020). The proposed mechanisms include the descent of the ventral structure (Soffers et al. 2015), asymmetric structural changes in the dorsal mesentery (Davis et al. 2008; Sivakumar et al. 2018), formation of duodenal and colonic flexures (Ginzel et al. 2021), and longitudinal growth of the small intestine (Kluth et al. 2003).

Recent studies have suggested the importance of mechanical forces in midgut morphogenesis. For example, Savin et al. (2011) demonstrated that mechanical forces arising from the differential growth between the midgut and mesentery drive the formation of small intestinal loops, where the mesentery grows slower than the midgut because of the bone morphogenetic proteins expressed in the mesentery (Nerurkar et al. 2017).

However, the roles of mechanics in the overall process of midgut morphogenesis are not fully understood. The concept of differential growth has been applied to understand the shape and deformation of biological tubes, such as arteries (Hannezo et al. 2012) and intestinal villi (Amar and Jia 2013). In the present study, we applied the continuum mechanics of differential growth to clarify the roles of mechanics in the protrusion, rotation, and retraction of midgut. The midgut was modeled as a hyperelastic tube with a Gaussian shape, and the differential growth of the midgut and mesentery was modeled using the spatial variation in spontaneous plastic deformation. We showed that protrusion, rotation, and retraction of the midgut can emerge sequentially because of temporal changes in differential growth.

**Fig. 2:**
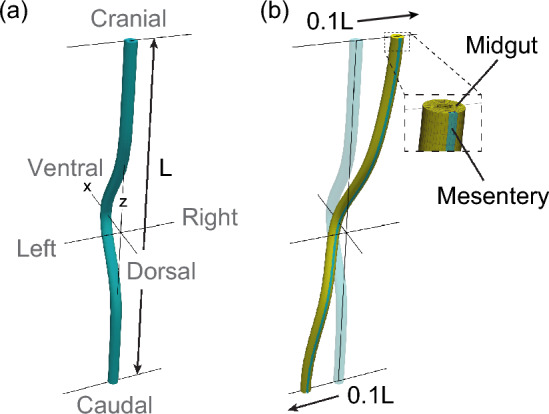
Midgut model. **a** A Gaussian shape is given by $x = 0.3Le^{-50(z/L)^{2}}$. **b** An initial displacement is given to the tube. The midgut and mesentery regions are shown in yellow and blue, respectively

**Fig. 3:**
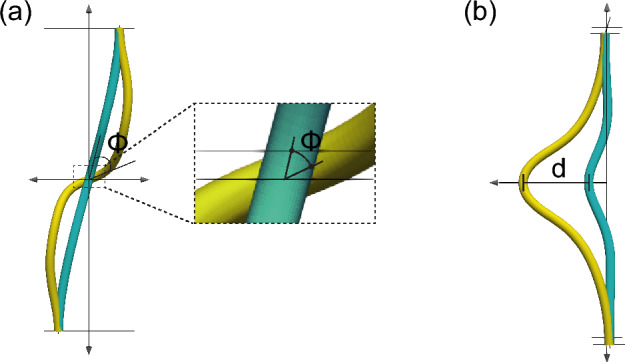
Evaluation of protrusion and rotation. **a** The amount of rotation $\phi$ and **b** the amount of protrusion *d*

## Methods

### Problem statement

The midgut was modeled as a compressible hyperelastic tube. A smooth curve is present in the ventral side of the midgut. Based on three-dimensional images and anatomical drawings of human, chick, and rat embryos (Southwell 2006; Kerwin et al. 2010; Soffers et al. 2015; Ginzel et al. 2021), the midgut shape was approximated as a Gaussian shape (Fig. 2a). This Gaussian shape was defined as the reference (stress-free) configuration. As the cranial limb of the midgut appears to be positioned to the right of the caudal limb before rotation (Kerwin et al. 2010; Southwell 2006), an initial displacement was applied to the tube, as shown in Fig. 2b (see also Appendix A for the effect of the initial displacement). Both ends of the tube were fixed to account for body length constraints.

According to Savin et al. (2011), the mesentery becomes very thin by E8, and its bending force is considered small compared to its tensile force. Therefore, the bending force of the mesentery was ignored, and the effect of the mesentery was modeled as a differential growth of the tube. Assuming that the region connected to the mesentery has the same physical properties as the mesentery, we defined a region with large Young’s modulus and slow growth rate (Fig. 2b). The differential growth between the midgut and mesentery was modeled by the spatial variation in spontaneous plastic deformation.

### Biological growth

A mathematical model for the growth of biological tissues was proposed in the context of continuum mechanics (Rodriguez et al. 1994). To describe the volumetric growth, the deformation gradient tensor $\varvec{F}$ was multiplicatively decomposed into growth-induced plastic deformation $\varvec{F}_{\textrm{g}}$ and hyperelastic deformation $\varvec{F}_{\textrm{e}}$ as follows:

1 $$\begin{aligned} \varvec{F} = \varvec{F}_{\textrm{e}} \cdot \varvec{F}_{\textrm{g}}. \end{aligned}$$

The growth-induced plastic deformation gradient tensor transformed the reference configuration into a new reference (stress-free) configuration:

2 $$\begin{aligned} \textrm{d} \varvec{X} = \varvec{F}_{\textrm{g}} \cdot \textrm{d} \varvec{X}_{0}, \end{aligned}$$

where $\textrm{d} \varvec{X}_{0}$ and $\textrm{d} \varvec{X}$ represent the infinitesimal vectors before and after plastic deformation, respectively.

To model unidirectional growth of the midgut, we considered the following deformation gradient tensor:

3 $$\begin{aligned} \varvec{F}_{\textrm{g}} = \varvec{I} + (\theta - 1) \varvec{T} \otimes \varvec{T}, \end{aligned}$$

where $\varvec{I}$ is the second-order identity tensor, $\theta (\ge 1)$ is the growth stretch, and $\varvec{T}$ is the unit tangent vector in the cylindrical direction.

### Hyperelastic deformation

The hyperelastic deformation gradient tensors transform the reference (stress-free) configuration after growth $\textrm{d} \varvec{X}$ into the final deformed configuration $\textrm{d} \varvec{x}$ as follows:

4 $$\begin{aligned} \textrm{d} \varvec{x} = \varvec{F}_{\textrm{e}} \cdot \textrm{d} \varvec{X}. \end{aligned}$$

The right Cauchy–Green deformation tensor is defined as follows:

5 $$\begin{aligned} \varvec{C}_{\textrm{e}} = \varvec{F}_{\textrm{e}}^{T} \cdot \varvec{F}_{\textrm{e}}, \end{aligned}$$

and the Green–Lagrange strain tensor is defined as follows:

6 $$\begin{aligned} \varvec{E}_{\textrm{e}} = \frac{1}{2}(\varvec{C}_{\textrm{e}} - \varvec{I}). \end{aligned}$$

We used a compressible Neo–Hookean model, wherein the strain energy density function is given as follows:

7 $$\begin{aligned} \psi _{\textrm{e}} = \frac{\lambda }{2}(\ln {J_{\textrm{e}}})^{2}+\frac{\mu }{2}(I_{\textrm{e}1}-3-2\ln {J_{\textrm{e}}}), \end{aligned}$$

where $I_{\textrm{e}1} = \textrm{tr} \varvec{C}_{\textrm{e}}$, $J_{\textrm{e}} = \textrm{det} \varvec{F}_{\textrm{e}} = \sqrt{\textrm{det} \varvec{C}_{\textrm{e}}}$. The lamé’s constants $\lambda$ and $\mu$ are defined as

8 $$\begin{aligned} \lambda&= \frac{G\nu }{\left( 1+\nu \right) \left( 1-2\nu \right) }, \end{aligned}$$

9 $$\begin{aligned} \mu&= \frac{G}{2\left( 1+\nu \right) }, \end{aligned}$$

where *G* is the Young’s modulus and $\nu$ is the Poisson’s ratio.

The variational formulation is given by

10 $$\begin{aligned} \delta W_{\textrm{ext}} = \delta W_{\textrm{int}}. \end{aligned}$$

The variation in the internal strain energy is given as

11 $$\begin{aligned} \delta W_{\textrm{int}} = \int _{V} \varvec{S}_{\textrm{e}} : \delta \varvec{E}_{\textrm{e}} \textrm{d}V, \end{aligned}$$

where

12 $$\begin{aligned} \varvec{S}_{\textrm{e}}&= \frac{\partial \psi _{\textrm{e}}}{\partial \varvec{E}_{\textrm{e}}} = 2 \frac{\partial \psi _{\textrm{e}}}{\partial \varvec{C}_{\textrm{e}}}, \end{aligned}$$

is the second Piola–Kirchhoff stress tensor and *V* is the volume in the reference configuration after the growth. The variation in the energy due to the external load is

13 $$\begin{aligned} \delta W_{\textrm{ext}} = \int _{S} \delta \varvec{v} \cdot \varvec{q} \textrm{d}S, \end{aligned}$$

where $\delta \varvec{v}$ is the virtual displacement, $\varvec{q}$ is the external load, and *S* is the surface in the reference configuration after the growth.

### Simulation conditions

The outer and inner radii of the tube were set to $R_{\textrm{o}} = 0.015L$ and $R_{\textrm{i}} = 0.005L$, respectively, where *L* is the distance between the ends of the tube. These values were defined with reference to the chick midgut at E8 (Savin et al. 2011).

The mesentery region is expressed by the sigmoid function as

14 $$\begin{aligned} f = \frac{1}{1+e^{2000\left( 0.15\sqrt{ (\frac{\xi^1 - \xi_{0}^1}{R_{\textrm{o}}}+1)^{2}+(\frac{\xi^2 - \xi_{0}^2}{R_{\textrm{o}}})^{2}}-0.035\right) }}, \end{aligned}$$

where (*ξ*^1^, *ξ*^2^) are the local coordinates in the cross section perpendicular to the central axis of the Gaussian-shaped tube, with the left-right axis being the *ξ*^2^-axis and the axis perpendicular to the *ξ*^2^-axis being the *ξ*^1^-axis, and $(\xi_{0}^1, \xi_{0}^2)$ are the center of each cross section (see also Appendix B for the effect of the sigmoid function). Using this sigmoid function *f*, the growth stretch and Young’s modulus are given by

15 $$\begin{aligned} \theta = \theta _{\textrm{mes}}+(\theta _{\textrm{gut}}-\theta _{\textrm{mes}})f, \end{aligned}$$

and

16 $$\begin{aligned} G = G_{\textrm{gut}} + (G_{\textrm{mes}} - G_{\textrm{gut}})(1-f), \end{aligned}$$

where $G_{\textrm{gut}}$ and $G_{\textrm{mes}}$ are the Young’s moduli of the midgut and mesentery regions.

We used isogeometric analysis (Hughes et al. 2005) with the Newton–Raphson method to solve the governing equations. The number of control points along the cylindrical direction is 131, the circumferential direction is 41, and the thickness direction is 9. We set the Poisson’s ratio to $\nu = 0.495$.

The amount of protrusion *d* is quantified as the displacement at the top of the Gaussian-shaped tube, as shown in Fig. 3. The degree of rotation $\phi$ is defined as the angle of rotation of the tube.

**Fig. 4:**
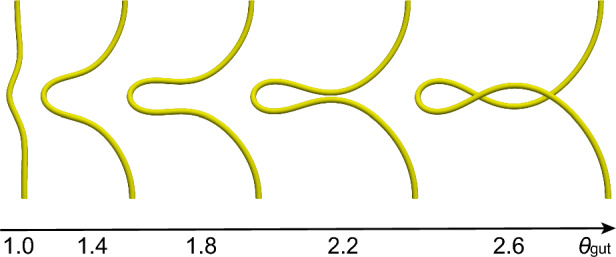
Tube shapes without differential growth for $1.0 \le \theta _{\textrm{gut}} \le 2.6$

## Results

### Chick embryo parameters

First, we considered midgut growth without mesentery. The deformation of a hyperelastic tube without differential growth was simulated. As shown in Fig. 4, the tube continued to protrude.

**Table 1:** Parameters extracted from chick embryos Savin et al. (2011)

Paramater	E5	E7	E7.2	E7.5	E7.8
$\theta _\mathrm {{gut}}$	1.0	1.21	1.32	1.48	1.63
$\theta _\mathrm {{mes}}$	1.0	1.21	1.24	1.27	1.29
$G_{\textrm{mes}}/G_{\textrm{gut}}$	7	7	7	7	7

**Fig. 5:**
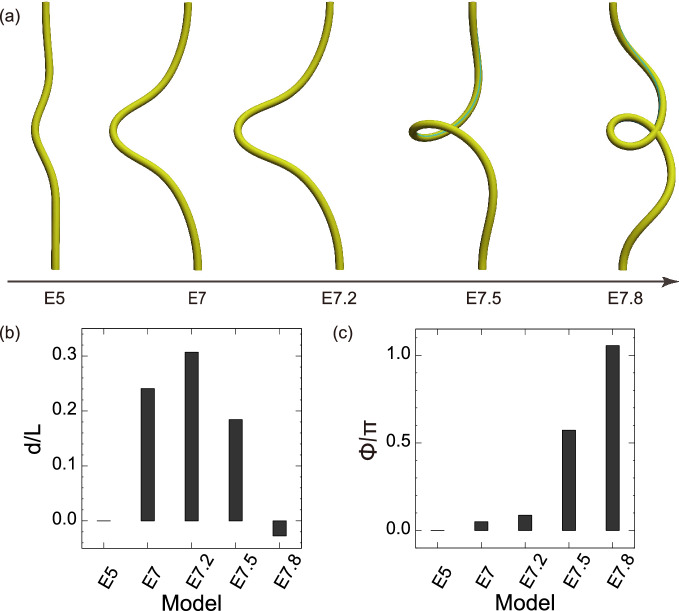
Simulation results with parameters extracted from chick embryos. **a** Time course of the tube shape. **b** Amount of protrusion and **c** amount of rotation

We next examined parameters extracted from actual chick embryos. Savin et al. (2011) reported that the chick midgut formed the first loop at embryonic day 5 (E5), and the lengths of the midgut and mesentery were almost equal until E7. Subsequently, the midgut rapidly increased in length, whereas the mesentery grew slowly. For example, the midgut and mesentery lengths were approximately 7.6 mm at E7, but increased to 10.9 mm and 8.3 mm, respectively, at E8. Based on these results, we assumed that the lengths of the midgut and mesentery increased at the same growth rate from E5 to E7 and at different growth rates from E7 to E8. They also reported that the Young’s moduli of the midgut and mesentery were approximately 4.54 kPa and 33.3 kPa, respectively, at E8. We used a constant value for the Young’s modulus ratio, $G_{\textrm{mes}}/G_{\textrm{gut}} = 7$ from E5 to E8. Parameters used in this section are summarized in Table 1, and the growth stretch at E5 is defined as $\theta _{\textrm{gut}} = 1.0$ (see Appendix C).

In chick embryos, the midgut begins to form a U-shaped midgut at E5 and the midgut rotates $90^{\circ }$ by E10 (Nerurkar et al. 2017; Huycke and Tabin 2018). Using the parameters in Table 1, the computational model successfully captured the developmental processes of midgut morphogenesis (Fig. 5). The Gaussian-shaped tube initially deformed into a U-shape, increasing the amount of protrusion to E7.2, without rotation. The tube then rotated approximately $90^{\circ }$ counterclockwise at E7.5. The tube continued to rotate, but retracted before forming multiple loops at E7.8.

**Fig. 6:**
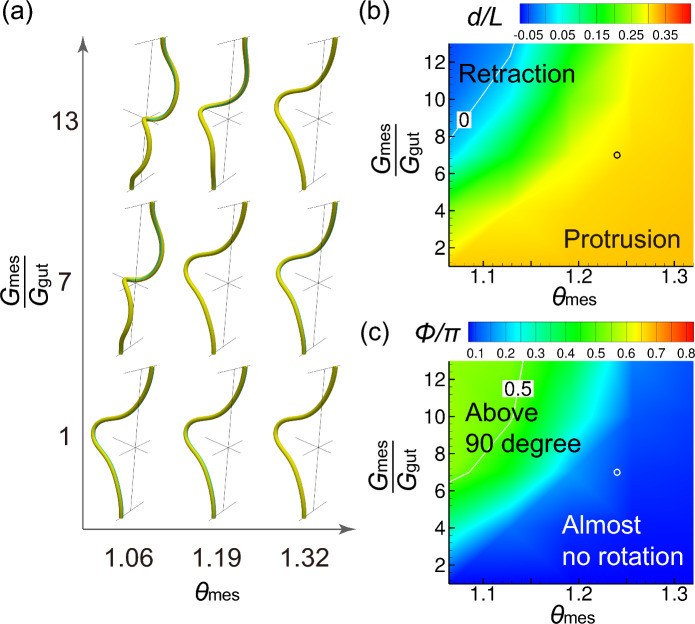
Effects of differential growth on tube protrusion. **a** Tube shapes for $\theta _{\textrm{gut}} = 1.32$. **b** Amount of protrusion and **c** amount of rotation. Circles indicate the parameter of the chick midgut at E7.2. White contour lines represent **b** $d/L = 0$ and **c** $\phi /\pi = 0.5$

**Fig. 7:**
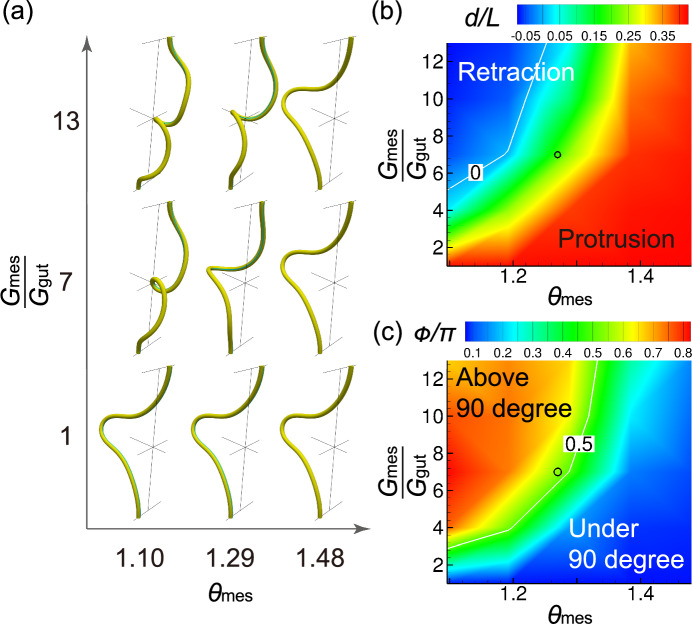
Effects of differential growth on tube rotation. **a** Tube shapes for $\theta _{\textrm{gut}} = 1.48$. **b** Amount of protrusion and **c** amount of rotation. Circles indicate the parameter of the chick midgut at E7.5. White contour lines represent **b** $d/L = 0$ and **c** $\phi /\pi = 0.5$

### Effect of differential growth

To identify the parameter space that enabled tube protrusion, a parametric study was performed on the E7.2 model. The growth stretch for the midgut was set at $\theta _\textrm{gut} = 1.32$, whereas that for the mesentery and Young’s modulus ratio was varied. For most of the parameters shown in Fig. 6, the tube is capable of forming a similar protruding U-shape without rotation. The amount of protrusion increased when $\theta _{\textrm{mes}}$ approached $\theta _{\textrm{gut}} (= 1.32)$. However, excessive differences in growth rate and Young’s modulus can cause the rotation and retraction of the tube, even at this early stage of morphogenesis ($\theta _\textrm{gut} = 1.32$).

We also examined the parameter space of the rotation process at $\theta _{\textrm{gut}}=1.48$ (E7.5). As shown in Fig. 7, a rotation of approximately $90^{\circ }$ ($0.4< \phi /\pi < 0.6$) is attained within a limited parameter space. Small differences in growth rate and Young’s modulus induced tube protrusion without rotation, whereas excessive differences caused tube retraction with undeveloped loops.

The shape of the midgut results from the balance of forces between the compressive force due to the rapid growth of the midgut and the pulling force due to the slow growth of the mesentery. When the pulling force is small, the midgut protrudes; when the pulling force is large, however, the midgut rotates and retracts.

### Comparison with human data

The digital human embryo atlas has been presented in previous studies (Kerwin et al. 2010; de Bakker et al. 2016). We compare our computational model with three-dimensional data obtained from the Human Developmental Biology Resource (HDBR) (Kerwin et al. 2010). According to these data, the human midgut appears to undergo $90^{\circ }$ rotation with a long protrusion.

**Fig. 8:**
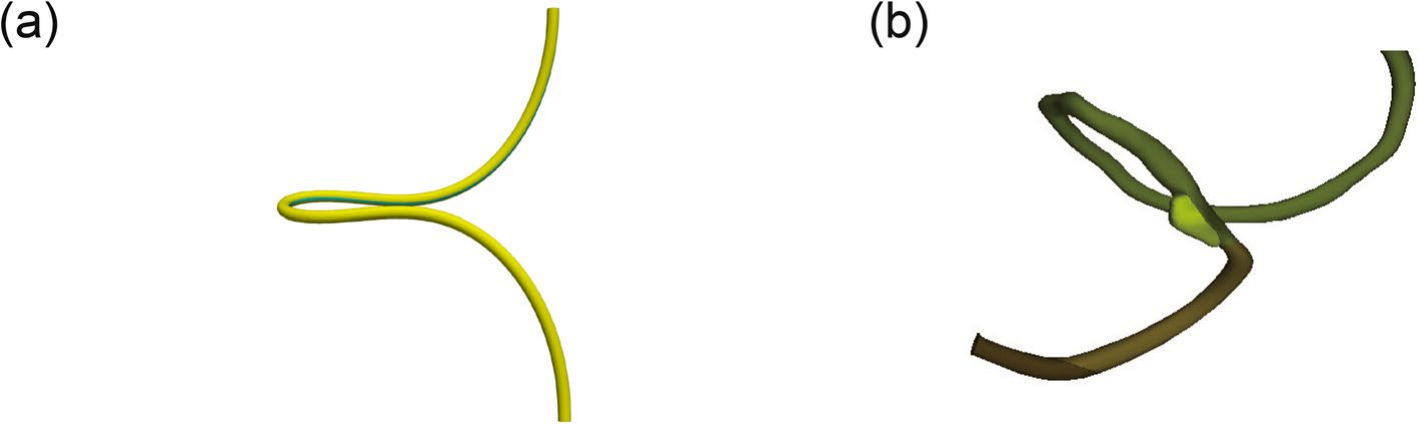
**a** Computational model for $G_{\textrm{mes}}/G_{\textrm{gut}} = 7$, $\theta _{\textrm{gut}}=2.0$, and $\theta _{\textrm{mes}}=1.87$. **b** Human midgut at CS 16. Embryonic samples from the 3D Atlas of Human Embryology were obtained and processed following protocols in de Bakker et al. (2016)

The lengths and Young’s moduli of the midgut and mesentery vary among species (Savin et al. 2011). In Figs. 6 and 7, $90^{\circ }$ rotation occurs at $\theta _{\textrm{mes}}/\theta _{\textrm{gut}} \sim 0.83$ for $\theta _{\textrm{gut}} = 1.32$, and $\theta _{\textrm{mes}}/\theta _{\textrm{gut}} \sim 0.87$ for $\theta _{\textrm{gut}} = 1.48$. As no data were available on the physical properties of the human midgut, we examined $G_{\textrm{mes}}/G_{\textrm{gut}} = 7$, $\theta _{\textrm{gut}} = 2.0$ and $\theta _{\textrm{mes}}/\theta _{\textrm{gut}} = 0.935$. Figure 8 compares the tube shapes of the computational model and human midgut at Carnegie stage (CS) 16. A $90^{\circ }$ rotation with a long protrusion was successfully reproduced using this computational model.

**Fig. 9:**
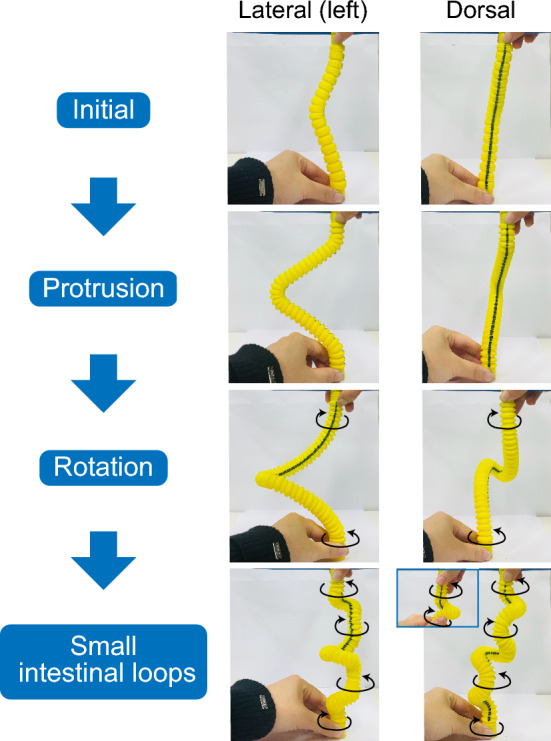
Gaussian-shaped tube develops a protrusion by compression-induced deformation. A less-growing mesentery (blue line) attempts to face inward, which causes tube twisting and results in midgut rotation. Repeated inward orientations cause the tube to twist everywhere, forming small intestinal loops

## Discussion

The continuum mechanics model successfully reproduced the sequential processes of midgut morphogenesis using the parameters extracted from chick embryos. Our results indicated that midgut protrusion, rotation, and retraction can emerge sequentially owing to temporal alterations in the differential growth of the midgut and mesentery.

The Gaussian-shaped tube protruded even without differential growth. This phenomenon was associated with the post-buckling behavior of the columns under compression. The tube increases in length while both ends are fixed; therefore, it cannot extend into the cylindrical direction, resulting in compression-induced deformation at the top of the Gaussian curve (Fig. 9). Midgut herniation occurs when the midgut elongates rapidly within a constrained abdominal space (Davis et al. 2008; Soffers et al. 2015; Huycke and Tabin 2018). Our continuum mechanics analysis supports this hypothesis.

Appropriate differential growth induced a $90^{\circ }$ rotation of the tube. If the tube has a smaller growing region at a bend, the region will attempt to face inward to minimize tensile forces (Fig. 9). The inward-facing orientation at the bend causes tube twisting, resulting in midgut rotation. The formation of the small intestinal loops can also be attributed to the same mechanism (Savin et al. 2011). The repeated inward orientations cause the tube to twist everywhere, as shown in Fig. 9.

Our results indicate that the external forces exerted in the ventral direction are not necessary for midgut herniation before $90^{\circ }$ of rotation. Conversely, tube retraction occurred subsequent to the $90^{\circ }$ rotation. The retraction of the midgut is known to occur after the formation of small intestinal loops. In biology, the expansion of the abdominal cavity has been considered to facilitate the midgut retraction (Soffers et al. 2015; Nagata et al. 2019). However, our model revealed an intrinsic tendency for retraction due to the mesentery. This suggests that external forces are necessary to maintain midgut herniation and prevent the retraction of the premature midgut during the formation of small intestinal loops. One potential candidate is the vitelline duct, which is connected to the midgut. A previous study (Chevalier et al. 2018) suggested the presence of pulling forces on the midgut through the vitelline duct. Another potential candidate is between the cranial and caudal limbs. During the formation of the small intestinal loops, the growth rate of the caudal limb becomes much slower than that of the cranial limb. The interplay between the mesentery (Savin et al. 2011; Nerurkar et al. 2017), vitelline duct (Chevalier et al. 2018), caudal limb, and abdominal cavity (Soffers et al. 2015; Nagata et al. 2019) may determine the initiation of midgut retraction in the abdominal cavity.

In this study, we have simplified this problem as a differential growth of a hyperelastic tube. However, in reality, the mesentery is a sheet-like tissue, and the bending, twisting, and contact dynamics of the mesentery may reduce the degree of midgut deformation. Coupling a three-dimensional tube with a two-dimensional shell is still computationally challenging, but this is an important future work toward more precise modeling. Another important future work is a modeling of mechanical feedback. Chevalier et al. (2018) demonstrated that tension from the vitelline duct promotes midgut growth. Therefore, the mechanical feedback is particularly important when the effect of the vitelline duct is considered.

In summary, we showed that temporal alterations in differential growth can induce protrusion, rotation, and retraction of the midgut because of mechanical problems. Although more realistic modeling, simulation, and experimental validation are needed, our computational model demonstrates the importance of the spatiotemporal control of mechanical forces in midgut morphogenesis. We hope that our results will be useful for future studies in embryology and tissue engineering.

## Figures and Tables

**Fig. 10:**
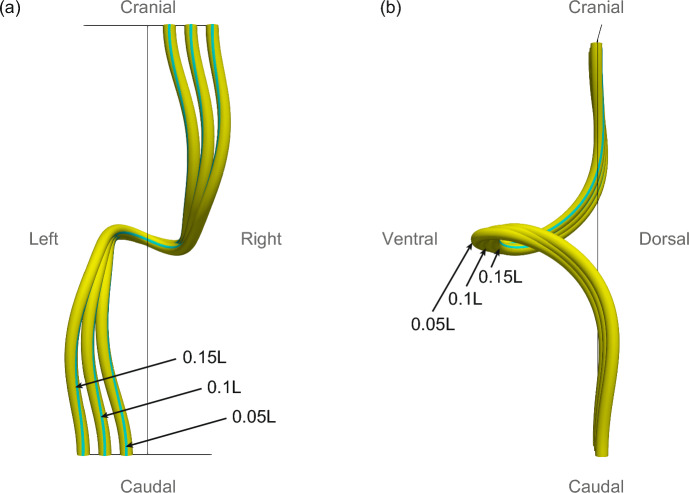
Effect of initial displacement. **a** The shape viewed from the dorsal side. **b** The shape viewed from the left side

**Fig. 11:**
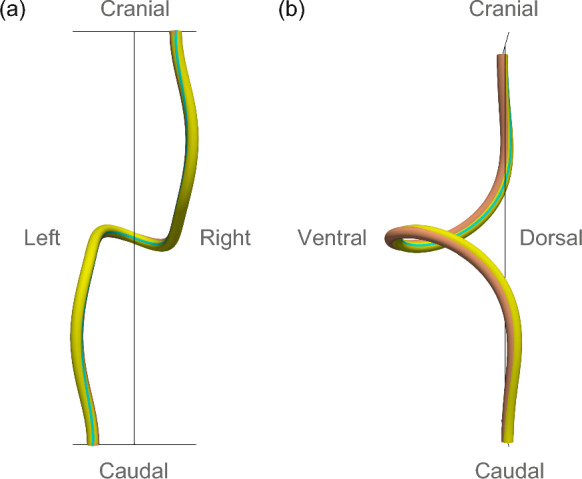
Effect of the gain of sigmoid function. **a** The shape viewed from the dorsal side. **b** The shape viewed from the left side. Yellow indicates the model with a gain of 2000 and orange indicates a gain of 1000

**Fig. 12:**
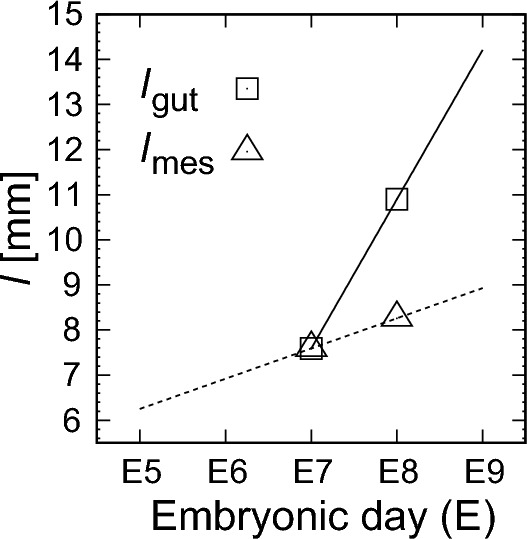
Estimated lengths of the midgut and mesentery based on Savin et al. (2011). The midgut and mesentery lengths at E7 and E8 were extracted from Savin et al. (2011)

## Data Availability

No datasets were generated or analyzed during the current study.

## Appendix A: Effect of initial displacement

In this study, an initial displacement of 0.1*L* was applied at both ends. To investigate the effect of the initial displacement, we compared the resulting shapes with initial displacements of 0.05*L*, 0.1*L*, and 0.15*L* for $G_{\textrm{mes}}/G_{\textrm{gut}} = 7$, $\theta _{\textrm{gut}} = 1.5$, and $\theta _{\textrm{mes}} = 1.3$. Qualitatively similar deformation patterns were obtained among these values of the initial displacements (Fig. 10).

## Appendix B: Effect of sigmoid function

The gain of sigmoid function controls the sharpness of the transition zone between the midgut and mesentery regions. In this study, the gain of sigmoid function was set to 2,000, which is sufficiently large to sharply separate the midgut and mesentery regions. We also examined a gain of 1,000 for $G_{\textrm{mes}}/G_{\textrm{gut}} = 7$ and $\theta _{\textrm{gut}} = 1.5$, $\theta _{\textrm{mes}} = 1.3$. The midgut shapes were nearly the same for gains of 1000 and 2000 (Fig. 11).

## Appendix C: Estimation of length of midgut and mesentery

Based on the fit curves in Fig. 3c in Savin et al. (2011), we assumed that the midgut and mesentery grew at the same rate until E7. We estimated the lengths of the midgut and mesentery from the curves as $l_{\textrm{gut}} = l_{\textrm{mes}} = 7.6$ mm at E7 and as $l_{\textrm{gut}} = 10.9$mm and $l_{\textrm{mes}} = 8.3$ mm at E8. We further assumed that the length increased linearly with the embryonic day, as shown in Fig. 12.
